# Understanding the Link between Inflammasome and Apoptosis through the Response of THP-1 Cells against Drugs Using Droplet-Based Microfluidics

**DOI:** 10.1021/acsomega.1c06569

**Published:** 2022-05-02

**Authors:** Elif Gencturk, Muge Kasim, Berna Morova, Alper Kiraz, Kutlu O. Ulgen

**Affiliations:** † Department of Chemical Engineering, Boǧaziçi University, Biosystems Engineering Laboratory, Istanbul 34342, Turkey; ‡ Department of Physics, Koç University, Sariyer, 34450 Istanbul, Turkey; § Department of Electrical and Electronics Engineering, Koç University, Sariyer, 34450 Istanbul, Turkey

## Abstract

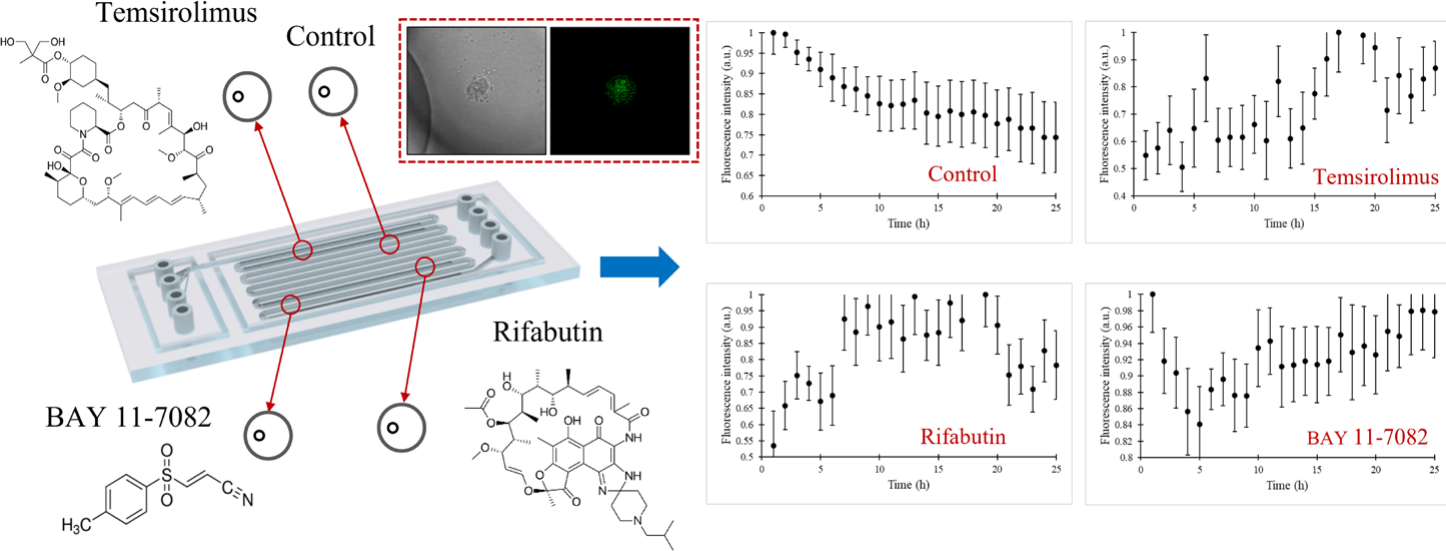

Droplet-based microfluidic devices are used to investigate monocytic THP-1 cells in response to drug administration. Consistent and reproducible droplets are created, each of which acts as a bioreactor to carry out single cell experiments with minimized contamination and live cell tracking under an inverted fluorescence microscope for more than 2 days. Here, the effects of three different drugs (temsirolimus, rifabutin, and BAY 11-7082) on THP-1 are examined and the results are analyzed in the context of the inflammasome and apoptosis relationship. The ASC adaptor gene tagged with GFP is monitored as the inflammasome reporter. Thus, a systematic way is presented for deciphering cell-to-cell heterogeneity, which is an important issue in cancer treatment. The drug temsirolimus, which has effects of disrupting the mTOR pathway and triggering apoptosis in tumor cells, causes THP-1 cells to express ASC and to be involved in apoptosis. Treatment with rifabutin, which inhibits proliferation and initiates apoptosis in cells, affects ASC expression by first increasing and then decreasing it. CASP-3, which has a role in apoptosis and is directly related to ASC, has an increasing level in inflammasome conditioning. Thus, the cell under the effect of rifabutin might be faced with programmed cell death faster. The drug BAY 11-7082, which is responsible for NFκB inhibition, shows similar results to temsirolimus with more than 60% of cells having high fluorescence intensity (ASC expression). The microfluidic platform presented here offers strong potential for studying newly developed small-molecule inhibitors for personalized/precision medicine.

## Introduction

Inflammation is the complex biological response of body tissues to harmful infections with pathogens, damaged cells, or irritants, and inflammation is a protective reaction involving immune cells, blood vessels, and molecular mediators. Arteriosclerosis, obesity, liver diseases, autoimmune diseases, Alzheimer’s disease, and cancer are the results of excessive or chronic inflammatory responses.^1,2^ Research on inflammation signaling pathways has accelerated the exploration of targets for drug discovery.^3^ In case of dangerous stimuli, macrophages take the major role in the inflammatory response. In vitro cancer, monocyte–macrophage differentiation, and macrophage-related physiological process studies are usually conducted with THP-1 cells.^4−6^

Innate and adaptive systems are two different immune systems of mammalian cells, and the innate immune system comes first during the protection against threats.^7^ The identification of detrimental cases within the cell is done by pattern recognition receptors (PRRs). Toll-like receptors (TLRs), C-type lectin receptors (CLRs), RIG-I-like receptors (RLRs), and nucleotide binding and oligomerization domain (NOD)-like receptors (NLRs) are the classes of PRRs.^8^ Among the NLR family, NLRP3 protein has a centrally located NOD motif, which is surrounded at the N-terminus by a pyrin domain for providing homotypic interactions with the adaptor protein, apoptosis-associated speck-like protein containing CARD (ASC - also called PYCARD). The activity of NLRP3 is mainly encountered in the cytosol of granulocytes, monocytes, dendritic cells, T and B cells, epithelial cells, and osteoblasts. This proves the theory that NLRP3 expression is necessary for the primary defense mechanism. Priming and activation are the two check-points of the NLRP3 inflammasome. In the priming step, NLRP3 and pro-IL-1β are transcriptionally induced, and in the activation step, the ASC-NRLP3 interaction is built.^9^ Under certain conditions, NLRP3 triggers the startup of cysteine protease caspase-1, and caspase-1 creates the pro-inflammatory cytokines interleukin (IL)-1β and IL-18 to form biologically active IL-1β and IL-18. The interaction between the NLRP3 and caspase-1 is ensured by ASC.^10^ IL-1β_ secretion was shown in various models of human monocytes and macrophages in a caspase-1/ASC/NLRP3-dependent pathway.^11^

ASC, a bipartite protein, includes two death domains, which are N-terminal pyrin (PYD) and C-terminal caspase recruitment (CARD). Due to its death-fold domains, it has a significant role in apoptotic cell death and inflammation. The conversion of procaspase-1 to active caspase-1 and maturation of interleukin-1 beta (IL-1β) and IL-18 (key proinflammatory cytokines) are conducted by ASC. This conversion and maturation events lead to pyroptotic cell death.^12^ Moreover, ASC is also known as a downstream target of methylation-induced gene silencing by DNA methyltransferase. ASC can be perceived as a probable tumor suppressor gene, and silencing of it might result in carcinogenesis in some tumors. The relation of ASC in cell death is driven by the conversion of caspase-1, and this ends with pyroptosis (a caspase-1 dependent cell death mechanism) through inflammasome formation. In 293T cells, ASC overexpression induced mitochondria or caspase 9 related apoptosis. ASC affects caspase 8 dependent apoptotic cell death. In human mammary epithelial cells, DNA damage or loss of extracellular matrix contact ended up with induced ASC expression leading to apoptosis.^13^

Allowing for low operation cost, short analysis times, small sample or reagent volumes, ease of separation, and detection with high resolution and sensitivity, microfluidic technology has served key roles in many fields including chemical synthesis, biological analysis, optics, and information technology. Many forms of microfluidic devices have been used in the literature for drug delivery,^14^ point of care diagnostics, organic synthesis, and microreactions.^15−17^ Microfluidic systems can be broadly classified based on their flow dynamics. Those that contain miscible liquids and operate in a single phase are called continuous flow, while two-phase systems that include immiscible fluids are defined as droplet-based.^18^

In this study, we examine the link between apoptosis and inflammasome processing in response to drugs used in tumor treatment. A droplet-based microfluidic platform is employed where the passive method is used to generate the droplets. THP-1 cells with the ASC gene tagged with green fluorescence are confined within the droplets containing several drugs (temsirolimus, rifabutin, and BAY 11-7082). We present a systematic way to understand the heterogeneous response of individual cells against drugs for deciphering cell-to-cell heterogeneity, which is an important issue in cancer treatment. The results obtained from the experiments are interpreted by statistical methods. The opportunity of live imaging of the same cells on an individual basis in a continuous manner makes the droplet microfluidics an attractive platform for future applications in medical therapeutics including the field of personalized/precision medicine.

## Materials and Methods

### Materials

THP-1 cells with GFP tagged ASC gene were kindly provided by Prof. Nesrin Özören from the Molecular Biology and Genetics Department of Boǧaziçi University. The COP made droplet generation and storage microfluidic devices (115 μm × 115 μm: *d* × *w*) (product code: Fluidic 719), fluidic interfaces with the type of mini luer, and tubings were purchased from Microfluidic ChipShop Company (Jena, Germany). Temsirolimus and rifabutin were kindly delivered by Pfizer. BAY 11-7082 was bought from Sigma-Aldrich (Taufkirchen, Germany). Novec 7500 and Krytox 157 FSH were purchased from 3M and DuPont, respectively. Necessary chemicals (RPMI 1640, fetal bovine serum (FBS), MEM Non-Essential Amino acid solution 100× (MEM-Nea), and penicillin–streptomycin 100× (Pen/Strep)) for medium preparation were acquired from ThermoFisher Scientific.

### Pre-Experiment Preparations

#### THP-1 Cell Culturing

Before THP-1 cell culture, all of the pieces of equipment were autoclaved and UV sterilized to prevent any contamination. RPMI complete medium containing 500 mL of RPMI 1640, 10% FBS (50 mL), 1% MEM-Nea (5.5 mL), and 1% Pen/Strep (5.5 mL) was prepared. The CO_2_ incubator was operated at 37 °C and 5% CO_2_ settings. THP-1 culture prepared from frozen stock was passaged 3 days apart in RPMI complete medium containing 20% FBS, 15% FBS, and 10% FBS, separately. In the last passage (10% FBS including RPMI complete medium), the cell became usable for experiments.

#### Oil and Drug Phase

Two-phase microfluidic devices were operated with two immiscible liquids to generate droplets. The oil phase (organic phase) was prepared with Novec 7500 and Krytox 157 FSH surfactant of 3%. The reason for adding Krytox 157 FSH to the oil phase is to reduce the surface tension between the two liquids. For the drug treatment experiments, temsirolimus (0.4 μg/mL), rifabutin (1.6 μg/mL), and BAY 11-7082 (10 μM) were used. In the droplet experiments, the concentrations of the drug solutions were adjusted to be twice the desired value since the drug medium and cell medium coming from different channels were mixed. Thus, the concentrations of temsirolimus, rifabutin, and BAY 11-7082 were 0.2 μg/mL, 0.8 μg/mL, and 5 μM during the experiments within the droplets, respectively. These concentrations were determined based on the previous works reported in the literature.^19−24^

#### Experimental Setup of the Microfluidic Device Platform and Operation

**Figure 1:**
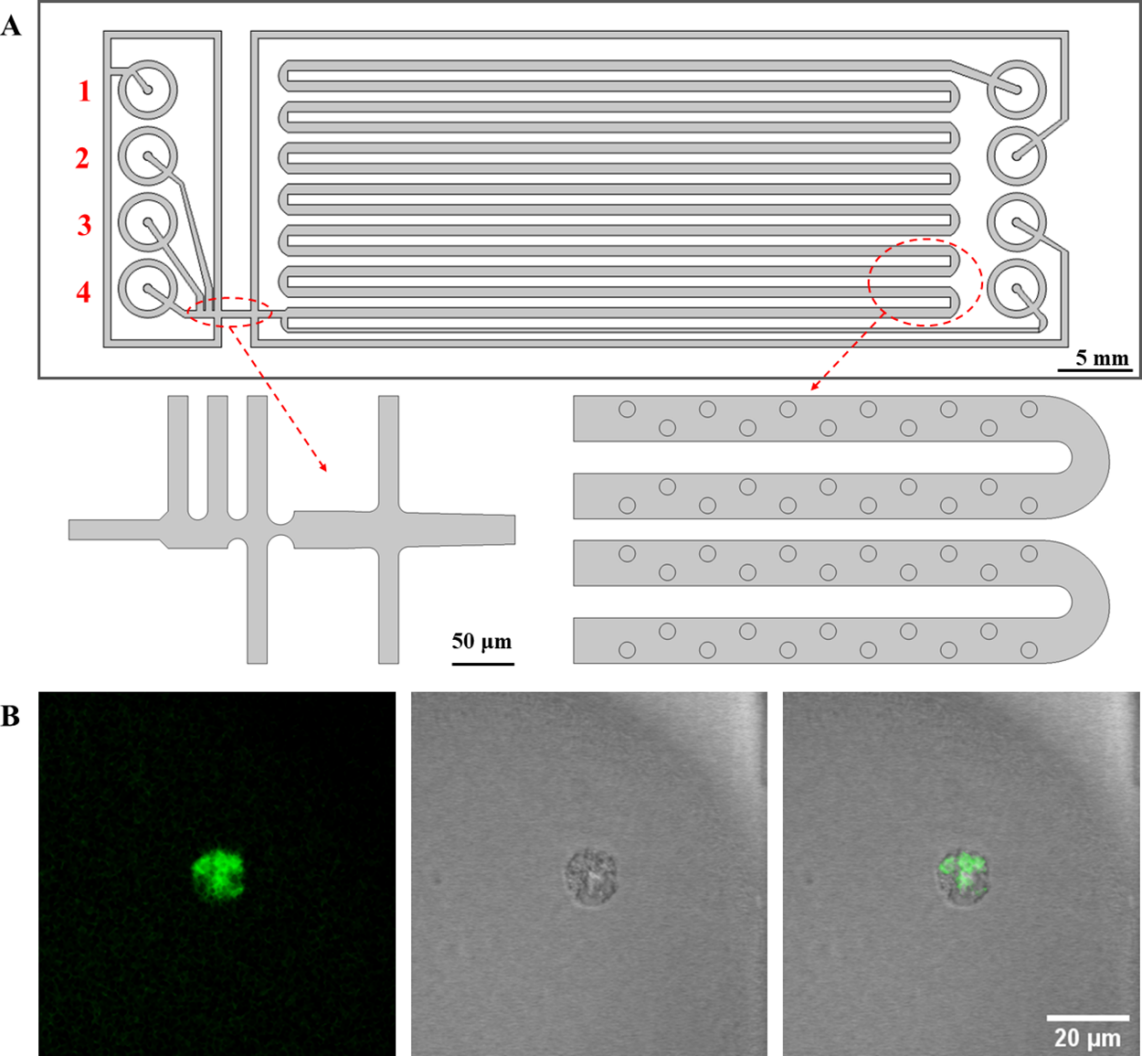
(A) Schematic representation of the microfluidic chip and focused versions of the droplet generation and droplet storage parts. (B) Fluorescence, bright-field, and merged microscopy images recorded from an exemplary THP-1 cell within a droplet.

The droplet generation and storage chip was placed on an inverted fluorescence microscope and kept within an environmental chamber providing 37 °C and 5% CO_2_. The ambient light was arranged to receive fluorescence microscopy images without noise. The chip has four inlets and four outlets. Inlets 1, 4, and 3 were used for continuous oil phase, THP-1 cells, and drug, respectively (Figure 1A). Oil, THP-1 cells, and drug-containing phases were filled into the syringes, and syringes were located on the syringe pumps. The syringe pumps were connected to the chip via PTFE tubings, and waste liquid was collected from the outlet of the chip. The continuous oil phase came from the upper and lower channels, and cells + drug came from the left channel (Figure 1A). Drug and cell-containing droplets were created at the junction of the channels and stored in the lines (Figure 1A). In the experiments, due to the channel configuration of the device (Figure 1A), droplets were generated by the flow-focusing method. Before cell loading, the chip was filled with the oil phase. When the chip was completely filled with oil, the cell and drug media began to be fed into the system at the same time. The THP-1 cell and drug-containing medium had 100 mL/h flow rates, while the oil had a flow rate of 600 mL/h.

Cells were encapsulated in the droplets with the drug, and the channels of the chip were filled with droplets (Figure 1B). When all of the channels were filled, the system was stopped and cell-containing droplets were marked. Bright-field and fluorescence microscopy images of the cells were recorded automatically with a time interval of 1 h, and the experiments lasted for 24 h. The expression of the ASC gene was followed by measuring its fluorescence intensity and by further processing using ImageJ.

### Imaging

Time-lapse live imaging was performed with an inverted confocal microscope (Leica DMI8) equipped with an incubation chamber. THP-1 cells were imaged at 37 °C with 5% CO_2_ with a frequency of 1 h per frame with 2 μm step size and 12 μm stack size in 1024 × 1024 pixel resolution at a specific position using an HC PL APO CS2 40× 1.30 NA oil objective. The expression of the ASC gene was followed by exciting the gene with 488 nm laser illumination and detection in the wavelength range of −500 to 573 nm. Gene expression was quantified by acquiring a z-stack with fixed gain and exposure settings for selected cells at all times, and the max slice projections were assembled from z stacks using ImageJ.

### Statistical Analysis

In order to interpret the variation of the experimental results, the method of randomized complete block design (RCBD) was used. This method is an extension of the paired *t*-test to situations where more than two operations must be analyzed. The observations can be represented by the linear statistical model
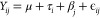
where μ is an overall mean, τ_*i*_ is the effect of the *i*th treatment, β_*j*_ is the effect of the *j*th block, and ϵ_*ij*_ is the random error. The RCBD follows the procedure that a sum of squares identity that partitions the total sum of squares into three components which are SS_Treatments_, SS_Blocks_, and SS_E_. The hypothesis is created as follows:
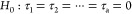

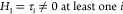


After computing the below variables, F-distribution was calculated. The null hypothesis is rejected at the α-level of significance if *f*_0_ > *f*_α,*a*–1,(*a*–1)(*b*–1)_.
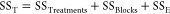

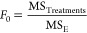


Fisher’s LSD method was used to reveal the specific distinctions. The value of LSD was calculated using
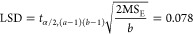


## Results and Discussion

THP-1 cells have similar morphology, secretory products, oncogene expression, and expression of membrane antigens to human monocyte cells. Since THP-1 cell has the ability to have a homogeneous population, biochemical studies can be done more easily. Thus, this cell line is more advantageous than native monocytes.^4^ In the following sections, the experimental results of the control and drug (temsirolimus, rifabutin, and BAY 11-7082) administered THP-1 cells (ASC:GFP) in the droplet-based microfluidic platform will be presented and discussed.

### The Behavior of THP-1 Cells in Droplets

**Figure 2:**
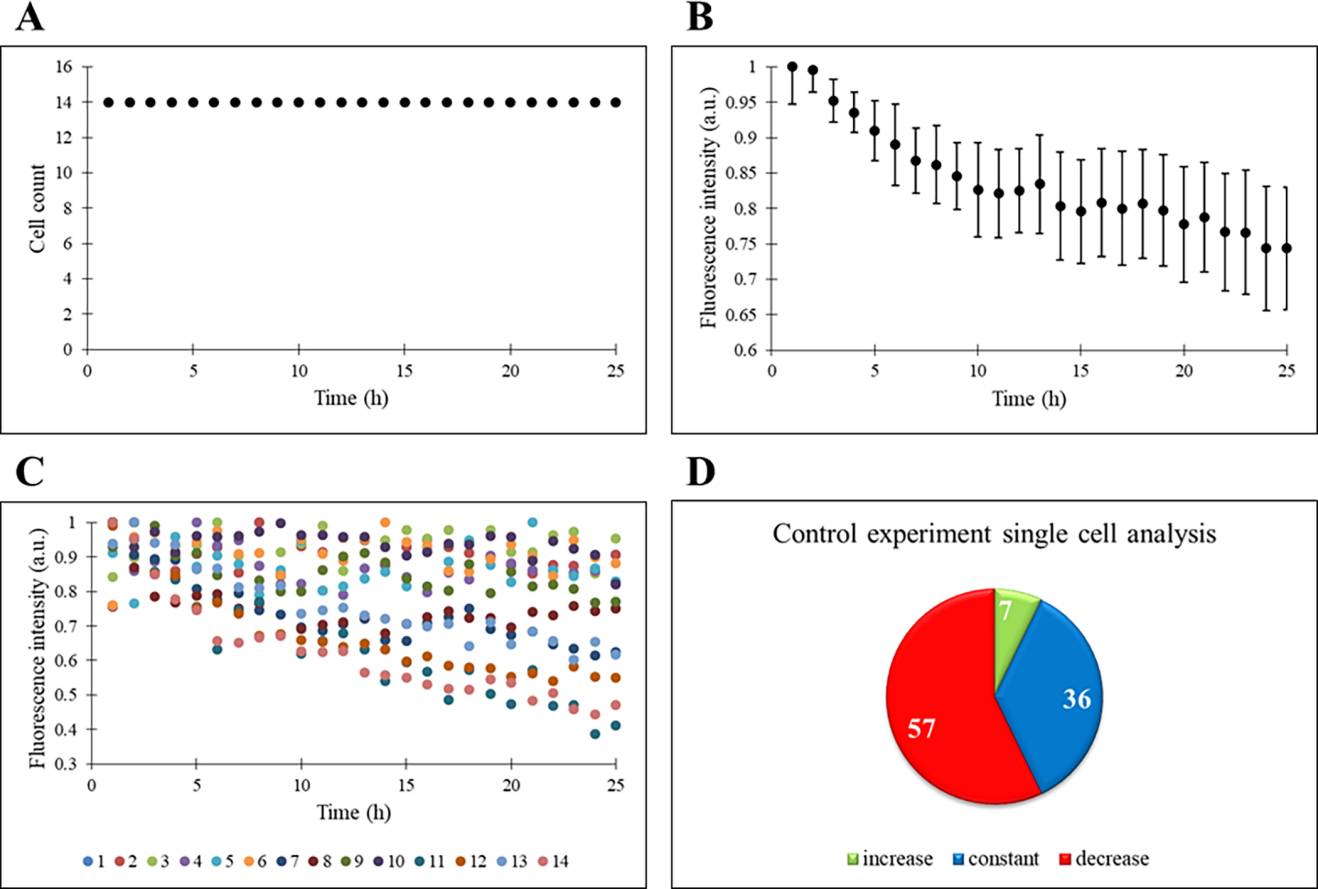
Control experiment. (A) Time profile of cell count. (B) Time profile of normalized fluorescence intensity (a.u.). (C) Time profiles of single cells’ normalized fluorescence intensity (a.u.). (D) Percentages of increase, constant, and decrease states of single cells’ fluorescence intensity.

THP-1 cells are confined in the droplets without drugs, and there is neither an increase nor decrease in the cell count for 60 h (only the first 24 h is shown in Figure 2A). The doubling time of THP-1 cells ranges from 19 to 50 h, but no proliferation is observed in the droplets and this might be the result of a limited nutrient environment. Similarly, Khorshidi and his colleagues performed a droplet-based experiment with HEK293T cells (doubling time around 24 to 30 h), and the cells survived up to 11 h in the droplets.^25^ In our experiment, the THP-1 cells sustained to live more than 24 h as tracked by their fluorescence intensity. Since THP-1 cells have GFP:ASC gene, the normalized fluorescence intensities of the cells are then analyzed (Figure 2B). There is a negative slope in the chart from the beginning of the experiment. In order for the ASC gene to be expressed, there must be a factor inducing the cell in the environment. However, since there is no drug/inhibitor treatment within the droplets, there is no stimulus for the cell to start inflammasome and hence a decrease in fluorescence intensity occurs. In addition, the standard deviation is calculated for the experiment, and due to its small value, the number of cells followed provided statistical accuracy.

In addition to the response of the cells as a population, single-cell responses are also given in Figure 2C,D. A change between 0.3 and 1.0 is observed in the amount of fluorescence intensity of the cells, and the decrease is noticeable through the end of the 24 h experiment. The increase, decrease, and constant tendency of the cells in the fluorescence intensity are determined by examining each cell one by one. Accordingly, if the slope of the trend line of the individual cell is up to 7 × 10^–4^ h^–1^, it is classified as constant, i.e., its fluorescence intensity is not changing throughout the course of the experiment. A slope above this value indicates an increasing trend for fluorescence intensity, and a negative slope with a lower value than 7 × 10^–4^ h^–1^ is interpreted as decreasing (Figure 2C). Here, 7.1% of the cells have an increasing trend, 35.7% of the cells have a constant trend, and 57.1% of the cells have a decreasing fluorescence intensity trend (Figure 2D). Compared to the number of cells with a reduction in fluorescence intensity, the number of cells with no change in fluorescence intensity throughout the experiment is also quite high. Moreover, fluorescence intensity has not ceased in any cell and this indicates the vitality of the cells. In resting cells, ASC can be found both in the cytoplasm and nucleus in the dissolved form.^26^ During the inflammation, one large micrometer-sized ASC speck per cell is detected and this creates the concentrating CASP1 activation sites.^26,27^ The generation of ASC specks is dependent on the inflammasome inducers.^28^ Since there is no inducer in this control experiment, the decrease in ASC fluorescence intensity is expected during the experiment.

### Effect of Tumor Treatment Drugs on THP-1 Cells in Droplets

#### The Behavior of THP-1 Cells in Response to Temsirolimus

Temsirolimus is a drug used for the treatment of several malignancies and solid tumors under the approval of the Food and Drug Administration (FDA). It is an ester derivative of rapamycin and inhibitor of the kinase mammalian/mechanistic target of rapamycin (mTOR). mTOR is considered as a probable target for cancer treatment. mTOR belongs to the phosphatidylinositol 3-kinase related kinase protein (PIKK) family, and it has a role in cell growth, cell proliferation, cell motility, cell survival, protein synthesis, and transcription. When the working mechanism of mTOR is disrupted, the probability of carcinogenesis increases and the abnormal function of mTOR results in several cancer types including the breast, lung, and pancreas.^29^

**Figure 3:**
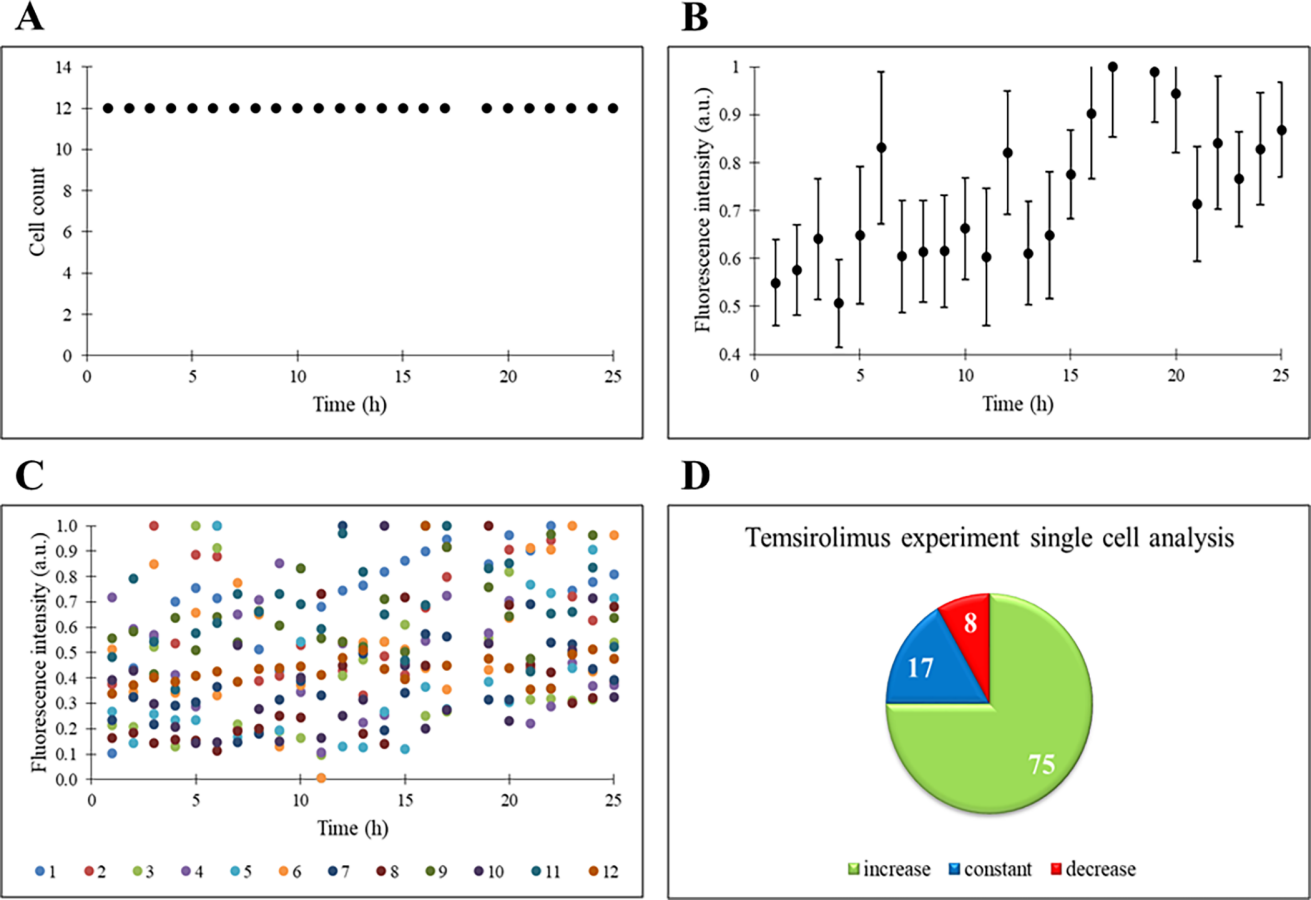
THP-1 cells are under the treatment of temsirolimus. (A) Time profile of cell count. (B) Time profile of normalized fluorescence intensity (a.u.) on the population basis. (C) Time profiles of single cells’ normalized fluorescence intensity (a.u.). (D) Percentages of increase, constant, and decrease states of single cells’ fluorescence intensity.

When temsirolimus is administered to THP-1 cells in droplets, the cell count does not change (Figure 3A) like in the control experiment. There is an increasing trend in the normalized fluorescence intensity in a range of 0.5–1.0 in Figure 3B. The small standard deviation in fluorescence intensity shows that the number of cells followed is sufficient to get meaningful and correct information on drug response. As stated previously, temsirolimus has an inhibitory effect on cells due to the interference of the mTOR pathway. Inflammasome should be triggered in cells under the treatment of drug, and the ASC-NLRP3 interaction is constituted. ASC oligomerizes into pyroptosomes, defined as perinuclear specs, and then autoproteolysis of procaspase-1 into caspase-1 occurs. The induction of IL-1β is realized by caspase-1, and pyroptosis starts.^9^ Since temsirolimus stimulates the apoptosis of cells^30^ and ASC plays an active role in cell death,^31^ an increasing expression (based on single cell fluorescence intensity) is seen in the experiment (Figure 3C). When the cells are examined individually, most of them show increased expression of the ASC gene; i.e., 75% of the cells show an increasing trend, 16.7% of the cells show a constant trend, and only 8.3% of the cells show a decreasing trend in fluorescence (Figure 3D). The presence of cells with decreased intensity may indicate that the drug is not effective on these cells, whereas the increase in fluorescence intensity in cells under the effect of temsirolimus shows that the drug is effective in apoptosis.

Temsirolimus triggers apoptosis in tumor cells via activation of caspases.^32^ This drug causes the suspension of protein synthesis that manages the proliferation, growth, and survival of tumor cells. Cells exposed to temsirolimus encounter a cell cycle arrest in the G1 phase, and it also decreases the production of vascular endothelial growth factor (VEGF) resulting in the inhibition of tumor angiogenesis.^33^ Due to the antiproliferative effect of temsirolimus, inhibition of necessary survival pathways, complex cell cycle effects, induction of apoptosis, and autophagy might occur.^34^

#### The Behavior of THP-1 Cells under Rifabutin Treatment

Innate immune responses are important for the pathology of infectious and inflammatory disorders such as acute abdominal inflammation, respiratory tract disorders, and cancers. Rifabutin is the semisynthetic derivative of rifamycin and a wide spectrum antibiotic. The working mechanism of rifabutin goes through the inhibition of bacterial DNA-dependent RNA polymerase. Thus, the initiation of RNA formation is suppressed and inhibition of RNA synthesis and transcription occurs.^35^

**Figure 4:**
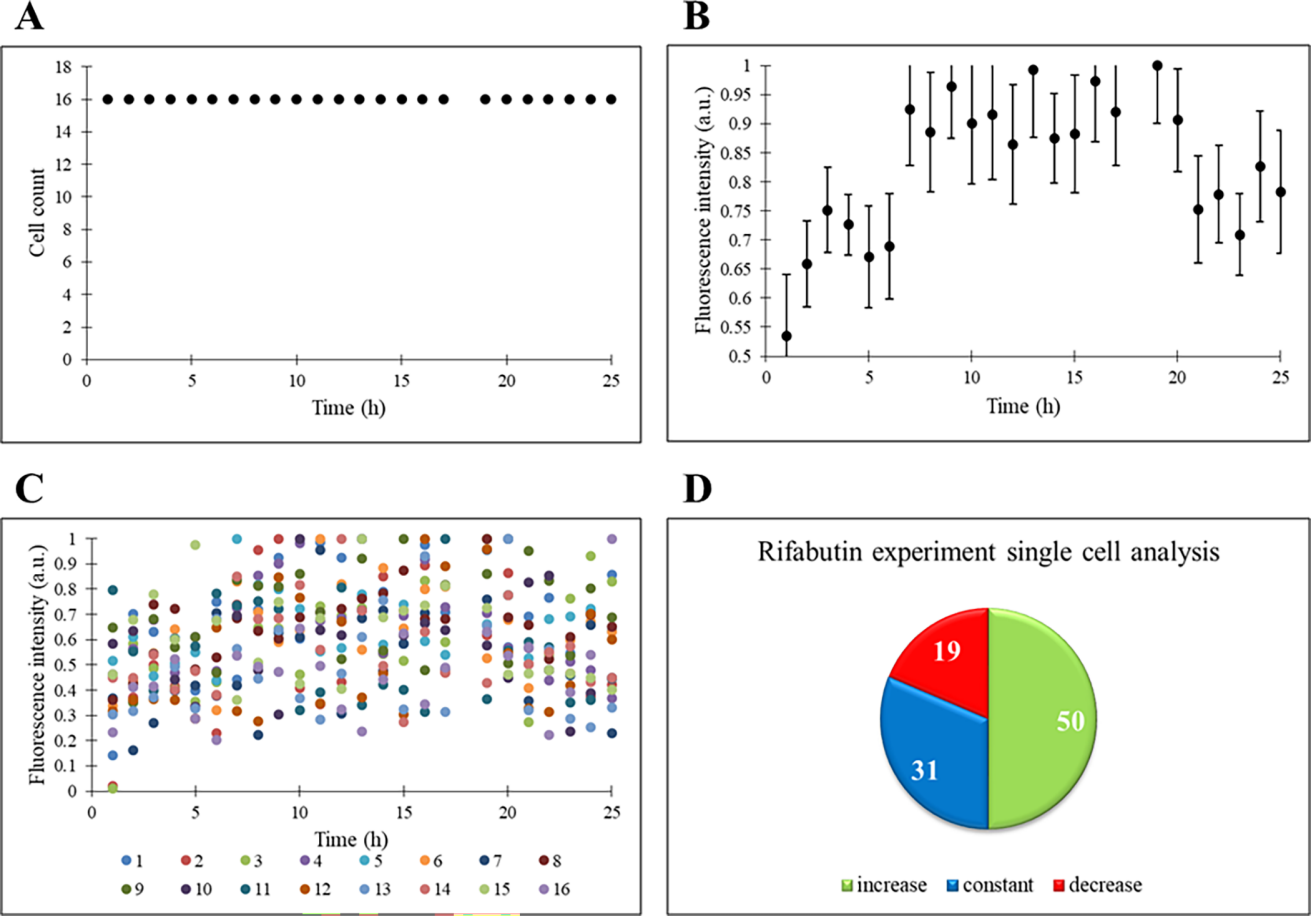
THP-1 cells are under the treatment of rifabutin. (A) Time profile of cell count. (B) Time profile of normalized fluorescence intensity (a.u.) of the population. (C) Time profiles of single cells’ normalized fluorescence intensity (a.u.). (D) Percentages of increase, constant, and decrease states of single cells’ fluorescence intensity.

In this section, rifabutin is administered to THP-1 cells. As in the control and temsirolimus experiments, the cell number does not change under rifabutin administration (Figure 4A). Normalized fluorescence intensities with error bars of the cells have an increasing trend at first, but there is a decrease in the intensity through the end of the experiment (Figure 4B). The small standard deviation indicates statistical accuracy in results. The single-cell responses under the treatment of the drug rifabutin show no big difference between the cells, and most of them have an almost similar reaction (Figure 4C). In our experiment, the expression of the ASC gene, triggered by rifabutin, first stops the proliferation and starts the apoptosis. Moreover, ASC is directly linked with CASP-3 and CASP-3 has a role in apoptosis. As stated previously, CASP-3 has an increasing level within the cells under inflammation conditions and there are several studies in the literature that promote this idea. According to these studies, it has been proven that inflammasome functions via oligomerization of the ASC, which is necessary for caspase-1 activation, release of IL-1β, induction of caspase-3 activation, and apoptotic cell death to occur eventually when a stimulant is present in the environment.^36−41^ Thus, the decrease in cell fluorescence intensity toward the end of the experiment may be due to CASP-3 resulting in cell death.

The rifabutin effect on the proliferation and apoptosis in lots of cancer cell lines was investigated in the literature, where rifabutin ceased proliferation and provoked apoptosis.^42^ Proliferating marker protein Ki67 and anti-apoptotic protein Mcl-1 had a decreasing concentration, whereas pro-apoptotic protein active caspase-3 had an increasing level within the cancer cells demonstrating the intervened proliferation and apoptosis effects of rifabutin.^42^

Finally, when looking at Figure 4D, increased ASC gene expression is seen in half of the cells, while the number of cells with decreasing fluorescence, that is, cells at the initiation of apoptosis, takes up to 20%. The number of cells with no change in ASC gene expression is around 30%. In these cells, either the function of the apoptosis-related ASC gene is terminated or the drug is not effective.

#### The Behavior of THP-1 Cells under BAY 11-7082 Treatment

The transcription of hundreds of genes, such as encoding for proteins comprising in immune regulation and also significant for cell survival, differentiation, and proliferation on non-immune cells, is governed by nuclear factor kappa light polypeptide gene enhancer in B cells (NFκB). Transcription factors of the NFκB family are activated and expressed by several stimuli such as proinflammatory cytokines and environmental stressors. The abnormal activity of NFκB results in many diseases such as tumor development and metastasis. Thus, inhibition of NFκB can be considered as an alternative option for tumor treatment, and anti-cancer drugs work for the inhibition of NFκB.^43^ BAY 11-7082 (BAY) is an anti-inflammatory drug and is used as an inhibitor of the expression of transcription factor NFκB.

**Figure 5:**
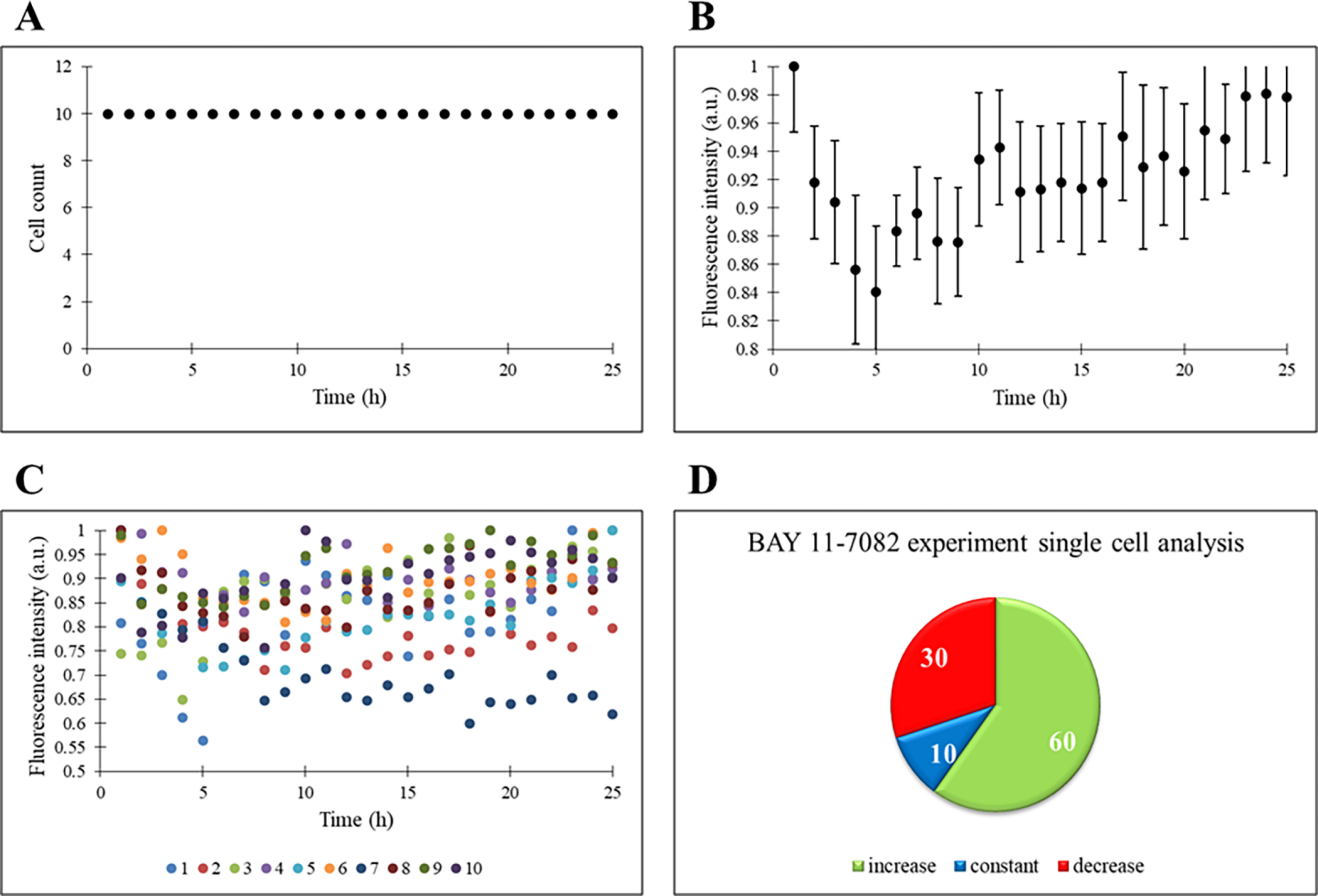
THP-1 cells are under the treatment of BAY 11-7082. (A) Time profile of cell count. (B) Time profile of normalized fluorescence intensity (a.u.) of the cell population. (C) Time profiles of single cells’ normalized fluorescence intensity (a.u.). (D) Percentages of increase, constant, and decrease states of single cells’ fluorescence intensity.

In response to BAY administration to THP-1 cells, the cell count did not change (Figure 5A). An increase in the ASC expression is detected on a population basis similar to the temsirolimus experiment (Figure 3B vs Figure 5B). In fact, a decrease in the fluorescence intensity is observed until the inflammasome complex becomes active (first 5 h), and then the increase in the reporter ASC expression, which is responsible for apoptosis, is normal in THP-1 cells whose cellular functions are inhibited by BAY 11-7082. When the single-cell response is considered, 60% of the cells show an increasing fluorescence intensity throughout the experiment (Figure 5C,D). In a study, HEK293 cells were treated with the BAY 11-7082 drug that caused the inhibition of TNF-like weak inducer apoptosis (TWEAK) triggered p100 processing (necessary for NFκB regulation) and TNF-induced phosphorylation and degradation of IκBα. The inhibition of these pathways resulted in the downregulation of NFκB.^43^ Due to the suppressed cell management capacity of NFκB, apoptosis within the cell might be triggered.

Under basal conditions, NFκB is located in the cytoplasm and it is inactivated by inhibitory IκB subunits. Proteasomal degradation of IκB is done via phosphorylation of itself, and this contributes to permitting nuclear translocation of NFκB and transcriptional activation of target genes.^44^ In case of inflammation, in response to cytokines and cellular stresses, IκB kinase (IKK) adds phosphate to IκB, and ubiquitination and proteolytic degradation occur. Then, NFκB activation and nuclear translocation materialize.^45^ The working mechanism of BAY 11-7082 starts with the inhibition of IKK, and this leads to the downregulation of NFκB. Inflammatory cytokine inhibition, heme oxygenase-1 induction, ICAM-1 activation suppression, reduction of ATPase activity of NLRP3 inflammasome, and the increase of neutrophil apoptosis are the processes where the drug BAY 11-7082 functions.^46,47^ In addition to its role in the activity of NLRP3, ASC is in collaboration with PYD families (ASC also contains PYD) such as PYPAF1 and PYPAF7, and they relate to NFκB activity or procaspase-1 activation.^48,49^ However, in the literature, ASC is described as a suppressor of NFκB activity,^50^ proposing that the ASC might have a dual role: the ASC effect on NFκB is dependent on the dosage of molecules.^51^ In detail, the duty of receptor interacting serine/threonine kinase 2 (RIP2) in the cell is to induce the NFκB signal. RIP2-deficient cells have reduced NFκB activation and cytokine generation. On the contrary, overexpressed RIP2 and caspase-1 in HEK293 cells show increased activity of NFκB proving that RIP2 is necessary for NFκB activation. Besides, when the ASC level is low, induction of NFκB continues. However, despite the existence of RIP2 and caspase-1, overexpression of ASC causes inhibition of NFκB. Therefore, ASC activity has a biphasic dose–response within the cells.^51^

### Statistical Analysis

In order to interpret the significance of the experimental results, the method of randomized complete block design (RCBD) is used, and SS_T_, SS_Treatments,_ and SS_Blocks_ are computed to be 25.688, 18.044, and 6.693, respectively. Thus, SS_E_ is calculated to be 0.951. When 95% confidence interval is considered, *f*_0.05, 3, 72_ is found to be 2.744^52^ and *f*_0_ is 11.02. Since *f*_0_ is greater than *f*_0.05, 3, 72_, there is a significant difference in the drug types as far as their effect on the cell is concerned.

RCBD indicated the difference among the experiments; therefore, Fisher’s LSD method is further used to reveal the specific distinctions. This method helps compare the mean of one group with the mean of another. The LSD value is estimated to be smaller than the mean difference of control vs temsirolimus, control vs BAY 11-7082, temsirolimus vs rifabutin, temsirolimus vs BAY 11-7082, and rifabutin vs BAY 11-7082 experiments but greater than the control vs rifabutin experiment. Hereby, temsirolimus is significantly different from the other three experiments. Since the LSD value is greater than the mean difference of control vs rifabutin, these two experiments do not differ so much. Fisher’s LSD method proves the accuracy of the experimental results, e.g., the fluorescence intensity values on a population basis under rifabutin and control experiments show a decreasing trend. In fact, rifabutin is a wide-spectrum antibiotic and is used mainly to treat mycobacterial infectious diseases rather than cancer. Due to rifabutin’s high activity against *Mycobacterium tuberculosis*, an etiological agent of tuberculosis, it is employed as a second-line anti-tuberculosis drug. Unlike rifampicin (first-line drug), rifabutin has limited interactions with antiretroviral drugs and this makes it a favorable drug for the treatment of human immunodeficiency virus (HIV) and TB infected patients.^53^ Pyroptosis is correlated with the control of intracellular pathogens, and activation of NLRP3 inflammasome is observed due to *M. tuberculosis* (MTB). With the help of IL-1β in protection against tuberculosis (TB), NLRP3 interferes in the early control of intracellular MTB replication.^54^ Being used for the treatment of MTB, rifabutin triggers the expression of ASC through NLRP3 inflammation and TB relationship.

## Conclusions

Fluid manipulation on the micro- or nano-scale is necessary in every field of today’s world technology to reduce the dimensions of equipment and machinery. One or two-phase microfluidic devices (continuous and dispersed) are operated as needed, and while the small molecule screening can be done via droplet-based devices, single-cell analysis can be done via continuous microfluidic devices. In this study, we are able to generate small droplets with the THP-1 cells inside. Several drug administrations (temsirolimus, mTOR inhibitor, rifabutin, anti-tuberculosis, and BAY 11-7082, NFκB inhibitor) are performed, and the cell response is monitored via the ASC gene. When there is no stimulus inducing the ASC gene in the environment, a continuously decreasing fluorescence intensity is observed in THP-1 cells. However, an increase in ASC gene expression is mainly observed in response to drug administration. It is evident from our microfluidic study that ASC plays a critical role in communication between the inflammasome and the apoptotic pathways.

The macromolecular signaling complex inflammasome is composed of different types of sensors activated in response to different stimuli, an adaptor protein apoptosis-associated speck-like protein containing a caspase recruitment domain (ASC), and caspase-1. Although programmed cell death pathways and inflammasome activation pathways are functionally different, recent literature information indicates that many signaling molecules known to regulate programmed cell death can also manipulate inflammasome activation. The interplay of well-known inflammasome NLRP3 with cell death systems is pointed in several infectious and inflammatory diseases, where the apoptosis-associated speck-like protein (ASC) mediates NLRP3 signaling. Lately, small-molecule inhibitors have been developed against individual ASC inflammasome components, namely, NLRP3, NLRP1, NLRC4, and AIM2. The neutralization of biological activities of dysregulated ASC inflammasome may offer new treatment strategies (design of new vaccines and immunotherapies targeting inflammasome) toward inflammatory diseases including cancer, and the microfluidic platform presented here can be used for unraveling cell-to-cell heterogeneity in pre-clinical studies of newly developed small-molecule inhibitors.
